# Selective cytotoxicity of a Vietnamese traditional formula, Nam Dia long, against MCF-7 cells by synergistic effects

**DOI:** 10.1186/s12906-016-1212-z

**Published:** 2016-07-16

**Authors:** My-Nuong Thi Nguyen, Thuy-Duong Ho-Huynh

**Affiliations:** Department of Genetics, Faculty of Biology, University of Science, 227 Nguyen Van Cu Street, District 5, Ho Chi Minh City, Vietnam

**Keywords:** Nam Dia Long, Traditional medicine, Selective cytotoxicity, Synergy, Apoptosis

## Abstract

**Background:**

Nam Dia Long (NDL) is a Vietnamese traditional formula used for the treatment of some chronic diseases, including cancers, but which lacks evidence-based support. We investigated the selective cytotoxicity of NDL on some tumor cell lines and possible interactions among its ingredients leading to the overall activity.

**Methods:**

Crude aqueous extracts of NDL, its ingredients including *Vigna radiata* (L.) Wilczek, *Vigna unguiculata* (L.) Walp. subsp. unguiculata, *Sauropus androgynous* (L.) Merr and different ingredient combinations were used for the treatment of MCF-7, Hep G2, NCI-H460 cells and normal fibroblasts. The IC_50_ of NDL on tumor and normal cells were determined by sulforhodamine B (SRB) assay and used to calculate a selectivity index (SI). Apoptosis induction activity of NDL was determined by acridine orange - ethidium bromide (AO-EB) staining, genomic DNA and cell cycle analysis. The combination index (CI) reflecting the types of interactions among ingredients was calculated based on the median-effect principle. Real-time cell growth monitoring by the xCELLigence system was used to determine the kinetic profile of the treated MCF-7 cells.

**Results:**

NDL exerted cytotoxicity on all tumor and normal cells, with the highest effect on MCF-7 cells. SI values for MCF-7, Hep G2 and NCI-H460 were 6.45, 1.61 and 1.29, respectively, indicating a high selective cytotoxicity of NDL toward MCF-7 cells. Profiles of cell death differed for MCF-7 cells and fibroblasts suggesting different mechanism of action of NDL toward these two cell types. The cytotoxicity of NDL against MCF-7 cells was due to apoptosis induction. NDL caused a cell cycle non-phase-specific effect on MCF-7 cells. CI indicated synergistic interactions among the ingredients leading to the overall activity of the complete formula. The real-time monitoring of MCF-7 cells growth after being treated with NDL and three-component combinations suggested that the presence of all ingredients was needed to reach the full cytotoxic activity. The growth kinetic profile of MCF-7 cells treated with different combinations also indicated a synergistic effect of all ingredients.

**Conclusion:**

NDL exhibited selective cytotoxicity toward MCF-7 cells. This effect probably resulted from synergistic interactions among the NDL ingredients. NDL should be explored for breast cancer treatment.

## Background

Vietnamese traditional medicine (VTM) is fully integrated into national healthcare and plays an important role in Vietnamese society. It is particularly indispensable for low-income people and those living in rural areas with limited or no access to Western medicine. Approximately 30 % of Vietnamese patients are treated with traditional medicine [1]. VTM has been strongly influenced by traditional Chinese medicine and shares the same holistic approach of Oriental medicine. The underpinning philosophy is to use mixtures of ingredients in one formulation to address different targets in the body to not only treat the disease but also regulate homeostasis. Therapeutic effects of multi-ingredient formulations result from interactions among these ingredients, which can be synergistic, additive or antagonistic [2]. A synergistic effect could have the advantage of reducing dose-related side effects while maintaining biological efficacy. Cancer, as a multifactorial disease, may benefit from this holistic approach.

NDL, a VTM formula, is empirically prescribed for some cancers and arthritis. It is composed of earthworm (*Pheretima aspergillum*), mung bean (*Vigna radiata* (L.) Wilczek), black bean (*Vigna unguiculata* (L.) Walp. subsp. unguiculata), and sweet leaf (*Sauropus androgynous* (L.) Merr.). Earthworm has antithrombotic, anticancer, wound healing, neuron regeneration, and anti-asthmatic activities [3–5]. Condensed tannins from black bean inhibit proliferation and migration of some tumor cell lines [6]. Flavonoids and polyphenols from mung bean seeds and sprouts have antidiabetic, antihypertensive, antitumor, antioxidant, anti-inflammatory, and immunomodulatory activities [7–9]. Sweet leaf, a widely-consumed leaf vegetable in Asia, exhibits cell toxicity through apoptosis and necrosis [10, 11]. There is no report on the combinatory effect of the complete formula for cytotoxicity and antiproliferative effects on tumor cell lines.

In this study, we used the combination index - isobologram equation, based on the median-effect principle, to quantitatively analyze the interactions among ingredients of NDL that generated cytotoxic effects on some tumor cell lines and normal fibroblasts. Based on IC_50_ values of each individual extract, we calculated the CI to evaluate the nature of interactions among these extracts.

This investigation had two purposes. First, we determined the cytotoxic and antiproliferative activities of NDL and its ingredients on some tumor cell lines and normal fibroblasts. Second, we analyzed the interactions of these ingredients leading to the ultimate cytotoxic activity of the formula.

## Methods

### Preparation of NDL and its ingredients

NDL was composed of four ingredients, all in the form of dried materials: earthworm (*Pheretima aspergillum* - Pa) 10 g, mung bean seed (*Vigna radiata* (L.) Wilczek - Vr) 20 g, black bean seed (*Vigna unguiculata* (L.) Walp. subsp. unguiculata - Vu) 20 g and sweet leaf (*Sauropus androgynous* (L.) Merr. - Sa) 40 g. All of these ingredients were identified by and obtained from the Traditional Medicine Hospital HCMC (Ho Chi Minh City, Vietnam). A water decoction was produced for clinical use in the Traditional Medicine Hospital HCMC. In this study, a quantity of NDL and each of the ingredients equivalent to ten times the normal dosage was soaked in water for 20 min, boiled for 3 h in an automatic herbal extractor to obtain aqueous extracts at a concentration of 1 mg raw material/mL, and lyophilized to obtain the dried powder. The extracts yield of NDL, Pa, Vr, Vu, and Sa was 0.08, 0.03, 0.04, 0.03 and 0.03 g/g of dried material, respectively. Dried powders were stored at −80 °C. Before use, powders of NDL, Pa, Vr, Vu, and Sa were dissolved in distilled water and 0.2 μm filter sterilized.

### Chemical fingerprint analysis by high performance liquid chromatography (HPLC)

The method was set up as described previously [12]. Extracts of NDL and the four ingredients (100 mg) were dissolved in HPLC grade methanol (100 mL) and filtered through a SPE filter to remove undissoved matters. The extracts were then eluted with methanol and recovered after methanol elimination by a rotary evaporator. The recovered extracts were dissolved in double distilled water at a concentration of 30 mg/mL for HPLC analysis. The analysis was carried out on a Shimadzu HPLC system with a SPA-M20A PDA detector. The separation was performed on a C-18 column (150 mm × 4.6 mm, 5 μm. Supelcosil™, LC-18). The mobile phase consisted of 1 % acetic acid in aqueous solution (A) and 1 % acetic acid in methanol solution (B). The gradient elution program was set as follows: 0 min, 90:10 (A:B); 24 min, 40:60 (A:B); 26 min, 0:100 (A:B); 31 min, 0:100 (A:B); 33 min, 90:10 (A:B); 40 min, 90:10 (A:B). The injection volume was 20 μl and the flow rate kept at 1.0 mL/min. Column temperature was set at 30 °C and the wavelength used for detection was 300 nm. All HPLC analyses were performed at least three times.

### Cell lines and cell culture

NCI-H460 (HTB-177), MCF-7 (HTB-22) and Hep G2 cells (HB-8065) were purchased from the American Type Culture Collection (Manassas, Rockville). Cells were cultured at 37 °C and 5 % CO_2_ in Eagle’s Minimal Essential Medium (EMEM) supplemented with 10 % (v/v) FBS (Sigma), 2 mM L-glutamine (Sigma), 20 mM HEPES (Sigma), 0.025 μg/mL amphotericin B (Sigma), 100 IU/mL penicillin G (Sigma), and 100 μg/mL streptomycin (Sigma). Cells used in this study were between passages 4 and 20.

### Primary fibroblast culture

The method was performed as previously described with some modifications [13]. Fresh adult foreskins were collected from a healthy male donor at the Department of Andrology in Binh Dan Hospital. The donor donating tissue gave written informed consent, and this study was approved by the ethical committee for biomedical research of the Vietnam National University, Ho Chi Minh city (Ref. 1387 QĐ-ĐHQG). The hospital obtained written informed consent from the donor, giving permission to collect his tissue and use for research purpose. Samples, stored in PBS, were cut into small pieces of 2–3 mm × 5 mm and incubated with 0.2 % (w/v) collagenase (Sigma) at 37 °C for 2 h. The cell suspension was then filtered through a cell strainer (70 μm). After centrifugation at 200 **×** g, 10 min, cell pellets were collected and resuspended in Dulbecco’s Modified Eagle Medium:nutrient mixture F12 (DMEM/F12) (Gibco). Cells were cultured in DMEM/F12 supplemented with 10 % (v/v) FBS, 20 mM HEPES, 0.025 μg/mL amphotericin B, 100 IU/mL penicillin G, 100 μg/mL streptomycin at 37 °C, 5 % CO_2_. Normal primary fibroblasts were identified through morphological observation and anti-vimentin/anti-cytokeratin 19 antibodies (Sigma) staining. Fibroblasts used in this study were between passages 2 and 5.

### SRB assay

The assay was performed as previously described with some modifications [14]. Cells, seeded at a density of 10,000 cells/well (MCF-7, Hep G2) or 7,500 cells/well (NCI-H460) in 96-well plates, were cultured for 24 h before being incubated with NDL or its ingredients at different concentrations for 48 h. Treated cells were fixed with cold 50 % (w/v) trichloroacetic acid (Merck) solution for 1–3 h, washed, and stained with 0.2 % (w/v) SRB (Sigma) for 20 min. After five washes with 1 % acetic acid (Merck), protein-bound dye was solubilized in 10 mM Tris base solution (Promega). Optical density values were determined with a 96-well micro-titer plate reader (Synergy HT, Biotek Instruments) at the wavelengths of 492 nm and 620 nm. The percentage of growth inhibition (Inh %) was calculated according to the formula: Inh % = (1-[ODt/ODc] x 100) %, in which ODt and ODc are the optical density value of the test sample and the control sample, respectively. Camptothecin (Calbiochem) was used as a positive control.

### Selectivity index (SI)

The degree of selectivity of the cytotoxic extracts was expressed as SI = IC_50_ in normal cells/IC_50_ in tumor cells.

### Isobologram analysis

The combinatory effect of NDL ingredients was determined based on the median effect principle analysis of Chou and Talalay (2006) [15]. Based on serial concentrations and the corresponding Inh % of NDL and the four ingredients, CI were calculated with the CompuSyn program (www.combosyn.com). CI < 1, CI = 1 and CI > 1 corresponded to synergistic, additive and antagonistic effects, respectively.

### Genomic DNA analysis

Cells were seeded in 10 cm dishes at a density of 2 × 10^6^ cells/dish for 24 h and incubated with NDL at the IC_50_ concentration. After 60 h incubation, cells were trypsinized, washed and lysed in buffer containing 10 mM Tris (pH 7.4), 10 mM EDTA, 0.2 % Triton X100. After centrifugation at 14,000 × g for 20 min, cells were incubated with RNase A (100 μg/mL) for 30 min at 37 °C. DNA was extracted by phenol:chloroform and precipitated in cold ethanol overnight at −20 °C. The DNA pellet was dissolved in TE buffer (10 mM Tris, pH 8.0; 0.1 mM EDTA), analyzed in 2 % agarose gel and visualised by EB staining.

### AO/EB double staining

Cells seeded in 6-well plates (2 × 10^5^ cells/well) were grown at 37 °C, 5 % CO_2_ for 24 h. Cells were then treated with NDL at the IC_50_ concentration. After 60 h treatments, cells were washed with PBS and stained with a solution of AO-EB (100–100 μg/mL). Cell morphology was observed by fluorescence microscopy.

### Cell cycle analysis by flow cytometry

MCF-7 cells were seeded at a density of 2 × 10^6^ cells/dish in 10 cm dishes. After 24 h growth, cells were incubated with NDL at the IC_50_ concentration for 24, 36, 48, or 60 h. Cells were then harvested, washed and fixed with 70 % ethanol for at least 2 h. The cell cycle profile was analyzed at 10,000 events by the BD Accuri C6 flow cytometer (BD Biosciences). Propidium iodide (5 μg/mL) was used for DNA labeling. Vinblastine (Sigma) was used as a positive control.

### xCELLigence assay

Real-time analysis of cell proliferation was performed with the xCELLigence system (Roche Applied Science). MCF-7 cells and fibroblasts were seeded at a density of 6 × 10^3^ cells to each well of 96-well E-plates and allowed to settle for 30 min at room temperature. E-plates were then placed in the RTCA DP in a humidified incubator at 37 °C with 5 % CO_2_. After 24 h, extracts of NDL or its ingredients at concentrations corresponding to the IC_50_ of the NDL formula were added to the wells. Cell index was measured every 2 min for 30 sweeps, then every 30 min for 96 sweeps and finally every hour for 100 sweeps. Plotted curves represented mean values of two measurements per well. The Inh % was calculated according to the formula: Inh % = (1-[Cit/Cic] x 100) %, where Cit and Cic were the cell index value of the test sample and the control sample, respectively, which were normalized to their cell index values at 24 h.

### Statistical analysis

Data were represented as means ± standard error (n ≥ 3). The Kruskal-Wallis test followed by the Dunn’s test were used for the analysis of NDL cytotoxicity on tumor and normal cells (Table 1). The Student’s *t* test was used for the cell cycle phases analysis of MCF-7 cells (Table 2) (Graphpad Prism software). A *p*-value <0.05 was accepted as a statistically significant difference.

## Results

### Cytotoxicity of NDL on three tumor cell lines and normal fibroblasts

**Fig. 1:**
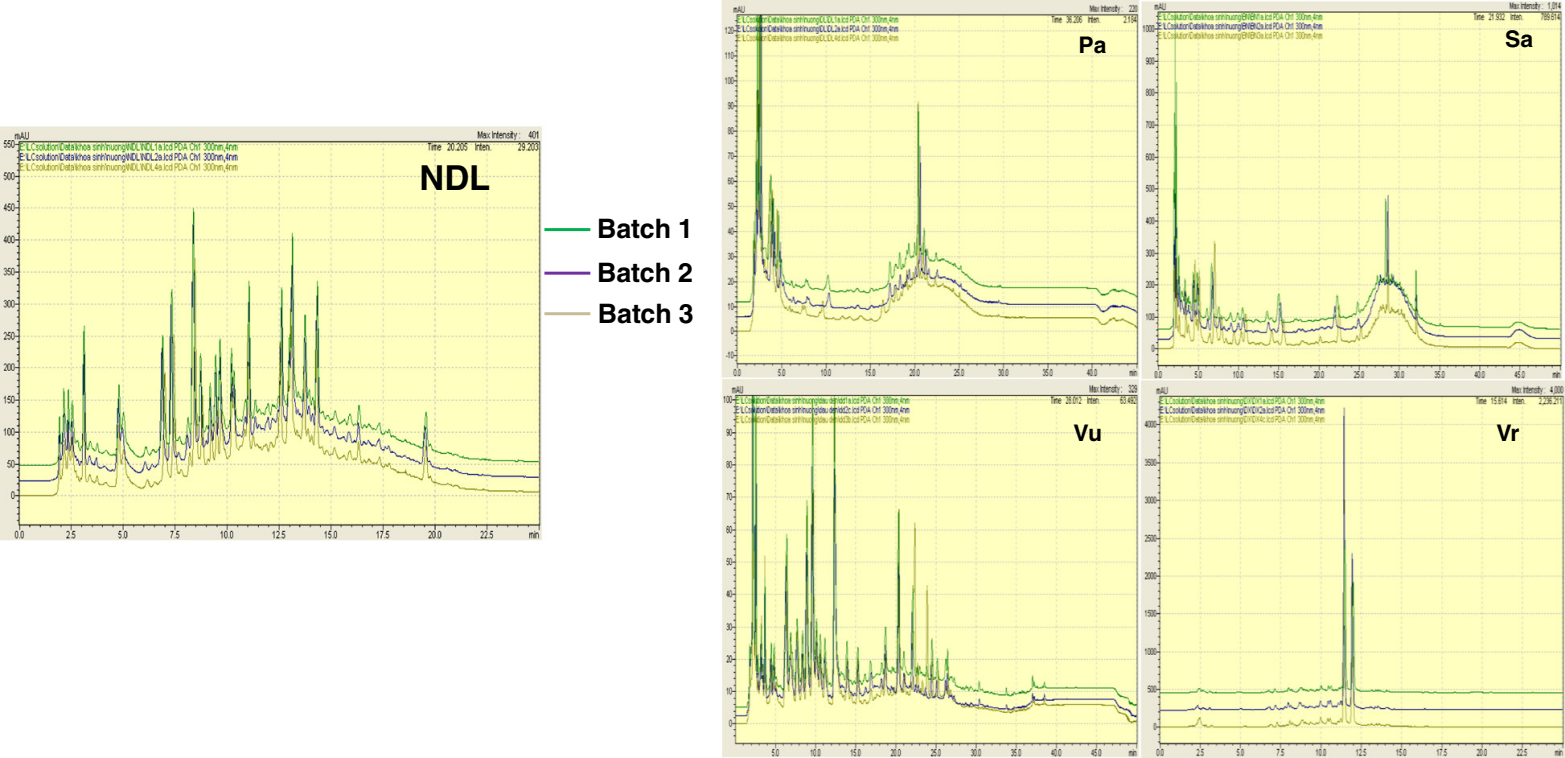
HPLC chromatograms of extracts from NDL and its ingredients. Experiments were repeated in three independent batches

The quality control of NDL and its ingredients was performed through the generation of fingerprint chromatograms by HPLC (Fig. 1). Fingerprint determination was repeated at least three times. Extracts of NDL, and its ingredients including earthworm (Pa), mung bean (Vr), black bean (Vu) and sweet leaf (Sa) showed similar LC profiles among the three batches tested, suggesting reproducibility in the preparation technology and the quality of the medicinal materials.

The cytotoxicity of NDL on MCF-7, Hep G2 and NCI-H460 cell lines and fibroblasts was determined by SRB assay. Among tumor cells, MCF-7 was the most sensitive to NDL, followed by Hep G2 and NCI-H460 (Table 1). Fibroblasts exhibited less sensitivity to NDL compared to tumor cells (Table 1). SI values determined for MCF-7, Hep G2 and NCI-H460 vs normal fibroblasts were 6.45, 1.61, and 1.29, respectively.

**Table 1:** Cytotoxicity of NDL on three tumor cell lines and fibroblasts

	MCF-7	Hep G2	NCI-H460	Fibroblast
IC_50_ (mg/mL)	0.63 ± 0.03 ***(#)	2.55 ± 0.19 *	3.15 ± 0.14	4.07 ± 0.39
SI	6.45 ± 0.52	1.61 ± 0.27	1.29 ± 0.15	1

Each value represents mean ± SD (n = 6). The statistical differences of IC50 value between cell lines were analyzed by the Kruskal- Wallis test followed by the Dunn’s test. *p<0.05 and ***p<0.001 vs fibroblast, while #*p*<0.001 vs NCI-H460

**Fig 2:**
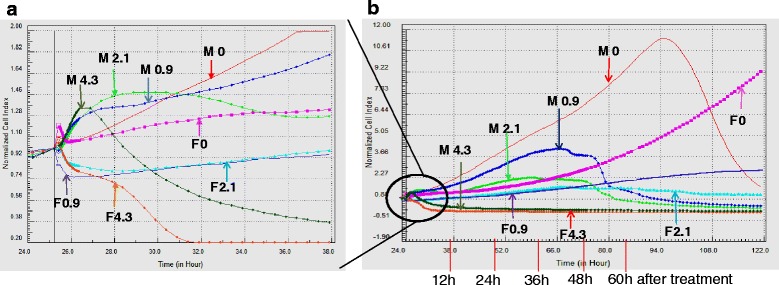
Real-time monitoring of cell growth for short-term (**a**) and long-term (**b**) on treatment of MCF-7 cells (M) and fibroblast (F)

We used the xCELLigence system for real-time monitoring of cell growth to further understand the selective cytotoxicity of NDL on MCF-7 cells. This real-time system recorded cell response to treatment in terms of proliferation and phenotypic changes, such as spreading or detachment. Based on the temporal effect of compounds on cell growth, the real-time analysis could eventually provide predictive mechanistic information. We performed analysis on MCF-7 cells, the most sensitive cell line, and fibroblasts treated with different concentrations of NDL. At a concentration of 4.3 mg/mL of NDL, MCF-7 cells and fibroblasts exhibited a drop of cell index value to near to 0, corresponding to total cell death or detachment. At concentrations below 4.3 mg/mL, short-term analysis of treated MCF-7 cells showed an immediate increase of cell index value over a few minutes (Fig. 2a), which was not observed on control cells. This rapid response of treated MCF-7 cells was probably due to physiological changes of the cells rather than proliferation. On the contrary, treated fibroblasts exhibited a cell index decrease during this period. Long-term monitoring of cell growth for more than 100 h showed a clear cytostatic profile of fibroblasts treated with NDL at concentrations under 4 mg/mL (Fig. 2b). During this period, MCF-7 cells treated with the same concentrations of NDL maintained a slight increase of cell index value until approximately 48 h after treatment, followed by a cell index decrease to 0 at about 90 h after treatment.

### NDL induced apoptosis on MCF-7 cells

**Fig. 3:**
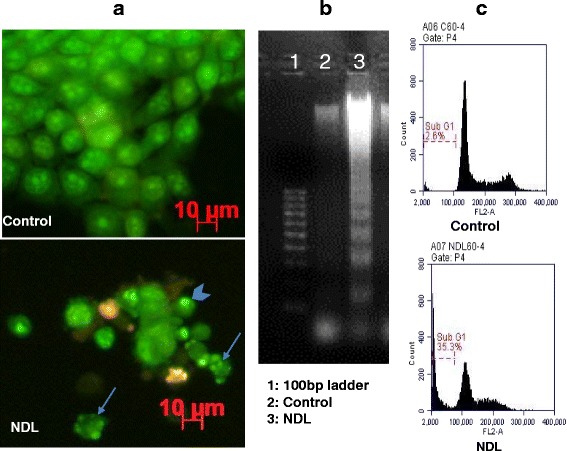
Apoptotic characteristics of MCF-7 cells treated with NDL. MCF-7 cells were treated with NDL at IC_50_ for 60 h. Morphological characteristics of treated cells were observed by AO-EB double staining. Arrowhead indicated nuclear condensation; full arrows indicated nuclear fragmentation (**a**). Genomic DNA fragmentation was analyzed by 2 % agarose gel electrophoresis (**b**). DNA histogram analyzed by flow cytometry revealed a Sub-G1 peak (**c**)

To determine the type of cell death, we performed genomic DNA analysis, cell cycle analysis by flow cytometry and fluorescence microscopy with AO-EB double-staining on MCF-7 treated with NDL. After 60 h treatment, MCF-7 cells exhibited some characteristics of apoptosis. Treated cells showed nuclear condensation and fragmentation (Fig. 3a). Genomic DNA analysis revealed a specific “DNA laddering” pattern (Fig. 3b). A sub-G1 peak was clearly observed on the DNA histogram from MCF-7 cells treated with NDL (Fig. 3c). Results showed significant reduction of the percentage of treated cells in G1, S and G2/M phases compared to control cells, which suggested a non-phase-specific cell cycle arrest (Table 2).

**Table 2:** Percentage of control and treated MCF-7 cells in cell cycle phases

		Percentage of cells in cell cycle phases			
		Sub-G1	G1	S	G2/M
24 h	Control	1.39 ± 1.37	34.68 ± 3.77	22.13 ± 0.46	26.36 ± 2.38
	NDL	0.62 ± 0.04	32.74 ± 0.73	23.76 ± 2.06	24.11 ± 2.11
36 h	Control	0.81 ± 0.63	47.26 ± 1.73	22.06 ± 2.11	19.56 ± 1.41
	NDL	3.18 ± 0.78*	49.08 ± 3.42	21.26 ± 0.22	16.93 ± 1.55
48 h	Control	0.64 ± 0.47	50.00 ± 1.49	21.04 ± 2.47	16.77 ± 1.25
	NDL	5.04 ± 0.90**	54.87 ± 2.26	15.97 ± 3.79	15.23 ± 6.21
60 h	Control	3.90 ± 2.88	59.27 ± 1.67	17.08 ± 0.68	14.67 ± 0.31
	NDL	35.38 ± 2.26***	40.47 ± 1.33***	13.05 ± 1.61**	8.50 ± 0.34***

Each value represents the mean ± SD (n = 3). The statistical differences between the treatment and control were analyzed by the two-tailed paired Student’s *t*-tests (* < 0.05, **p < 0.01; ***p < 0.001)

### Interactions among ingredients leading to the ultimate cytotoxic effect of NDL formula

**Fig. 4:**
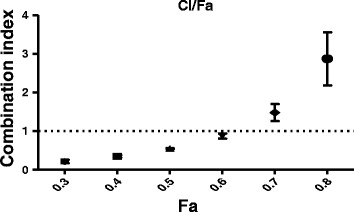
Fa-CI plot of NDL in MCF-7 cells. A CI of 1.0 (dashed line) reflected additive effects, whereas CI values greater than 1.0 and less than 1.0 indicate antagonism and synergy, respectively

To identify the type of interactions among ingredients leading to the cytotoxicity of the complete formula, we determined CI values based on the median effect principle method of Chou and Talalay [15]. First, the Inh % on MCF-7 cells, corresponding to the effect levels (or Fa (fraction affected)) of different concentrations of NDL and the four ingredients was determined by SRB assay. The range of Fa determined was from Fa = 0.3 (IC_30_) to Fa = 0.8 (IC_80_). CI values corresponding to different Fa were then calculated with the CompuSyn program and the Fa - CI plots were generated (Fig. 4). CI values < 1 were observed for Fa 0.3–0.6. CI > 1 were determined for Fa = 0.7 and 0.8. All experiments were repeated three times.

**Fig. 5:**
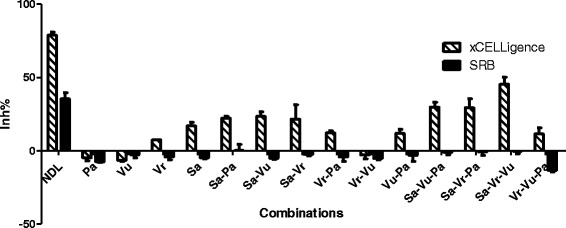
Cytotoxicity of NDL, each ingredient or two-, three-ingredient combinations on MCF-7 cells

To analyze the contribution of each ingredient in NDL cytotoxicity, NDL at the concentration of IC_50_, each ingredient, ten different two- and three-ingredient combinations were prepared for MCF-7 cells treatment. All the ingredients, in separated or mixed form, had a concentration equivalent to the IC_50_ concentration of NDL. NDL exerted a clear inhibitory effect that was not observed for each ingredient (Table 3, Fig. 5). CI values calculated for NDL, two-, and three-ingredient combinations showed that NDL and six combinations (Sa-Vr, Vr-Pa, Vu-Pa, Sa-Vr-Pa, Sa-Vr-Vu, Vr-Vu-Pa) benefited from synergistic interactions of the components with NDL exhibiting the strongest effect. Interestingly, six out of the seven combinations exhibiting synergistic effects contained mung bean (Vr) (Table 3).

**Table 3:** Cytotoxicity of NDL, each ingredient and two- or three-ingredient combinations on MCF-7 cells

	Inh %		CI of Fa = 0.5
	xCELLigence	SRB	
NDL	78.88 ± 2.17	35.41 ± 4.17	0.55
Pa	−4.84 ± 2.02	−7.48 ± 0.38	
Vu	−6.62 ± 0.60	−2.76 ± 2.13	
Vr	7.39 ± 0.21	−4.32 ± 1.94	
Sa	17.06 ± 2.36	- 4.77 ± 0.91	
Sa-Pa	22.21 ± 1.46	0.12 ± 4.39	1.64
Sa-Vu	23.55 ± 2.92	−5.10 ± 0.76	1.35
Sa-Vr	21.56 ± 9.70	−2.63 ± 1.04	0.41
Vr-Pa	12.31 ± 1.36	−4.26 ± 3.04	0.51
Vr-Vu	−2.96 ± 2.55	−5.36 ± 0.96	1.21
Vu-Pa	11.73 ± 2.88	−3.13 ± 4.10	0.92
Sa-Vu-Pa	29.80 ± 3.16	−1.07 ± 1.79	1.17
Sa-Vr-Pa	29.44 ± 5.95	−0.81 ± 2.23	0.78
Sa-Vr-Vu	45.31 ± 4.75	−0.30 ± 1.94	0.76
Vr-Vu-Pa	11.49 ± 4.13	−13.47 ± 0.89	0.73

MCF-7 cells were treated with NDL at the IC_50_ concentration, ingredients and the combinations at concentrations equivalent to the IC_50_ of NDL for 48 h. Cytotoxicity was measured by xCELLigence and SRB assay. CI of the two- and three-ingredient combinations were calculated by the Chou-Talalay method

**Fig. 6:**
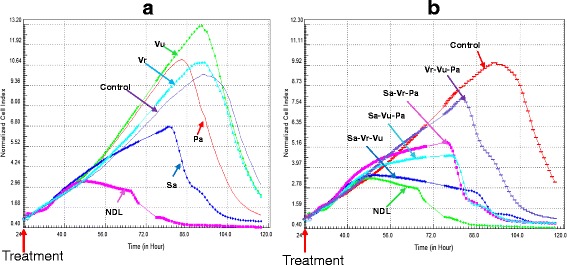
Real-time monitoring of cell growth of MCF-7 treated with NDL, each ingredient and three-ingredient combinations. MCF-7 cells were treated with NDL at IC_50_ concentration and each ingredient at concentration equivalent to the IC_50_ of NDL (**a**), and all three-ingredient combinations (**b**)

Real-time monitoring of cell growth showed different kinetic profiles of MCF-7 cells treated with NDL and with combinations lacking one of the four ingredients (Fig. 6). The kinetic profile of cell growth can be divided into three phases. During the first phase that lasted about 20 h after treatment, MCF-7 cells treated with NDL, the separate ingredients and all three-ingredient combinations exhibited a similar cell index increase as the control cells. During the second phase, the ingredients alone and the combination Vr-Vu-Pa showed a similar kinetic profile to the control cells whereas NDL and the three other three-ingredient combinations exhibited a cytostatic profile. The percentage of the inhibitory effect of NDL was remarkably higher than any of its ingredients (Fig. 6a) or combinations (Fig. 6b). Combinations without sweet leaf (Sa) had a delayed and more diminished cytotoxicity than other combinations. Combinations without earthworm (Pa) showed a significant diminution of inhibitory effect compared to the complete formula, but this was not as pronounced as for the other three-ingredient combinations. The combination without sweet leaf (Sa) gave the lowest inhibitory effect compared to other combinations (Fig. 6b). The onset of the last phase that was characterized by a decrease cell index varied depending on the combinations and ended by a cell index value near to 0.

## Discussion

Cancer is a world-wide health issue, with growing incidence due to increased industrialization and changes of environmental conditions, including lifestyle. Sophisticated methods based on chromatographic separation are used for isolation of pure anticancer substances from natural sources. On the other hand, combinatorial chemistry generates numerous synthetic chemicals for anticancer purposes. However, only a few clinically significant chemotherapeutic agents were discovered through large screening projects, and the development of synthetic drugs that have both efficacy and low toxicity were relatively inefficient, though extremely costly [16]. A totally different approach, represented by various traditional medicine systems, including VTM, assumes that maximal therapeutic efficacy can be obtained through complicated interactions among all ingredients of a formulation. These interactions can be synergistic, additive or antagonistic to each other aiming at reducing adverse side-effects, or enhancing and prolonging the therapeutic process [1, 17]. Since cancer is a multifactorial and highly heterogeneous disease, polychemotherapy, such as multi-ingredient formulations, is considered to be more active than single agent treatment [2, 17]. In this study, NDL and its four ingredients were prepared as aqueous extracts, since in VTM the most frequent administration form is aqueous decoction. We used the median-effect principle of Chou and Talalay to quantify the effect of combined ingredients from NDL. The advantage of using this method was that the determination of synergism, antagonism or additive effects does not require an understanding of the mechanism of action of the complete formula or its separate ingredients [15]. NDL exhibited significantly higher activity compared to the separate ingredients. Cell growth inhibition by NDL and the four combinations, each lacking one ingredient, showed that all ingredients were necessary for the overall cytotoxic effect, even though they possessed cytotoxicity at different levels. Sweet leaf (Sa) seemed to be the leading component for cytotoxicity of NDL, in agreement with previous reports showing extracts from *S. androgynus* induced apoptosis and necrosis on fibroblasts and endothelial cells [10, 11]. Earthworm (Pa), with its low cytotoxicity, possibly contributed through another activity. CI values calculated for NDL concentrations of IC_30_ to IC_60_ values were less than 1, indicating strong and stable synergistic interactions among all ingredients. At the high concentration of the IC_80_, the NDL ingredients showed antagonistic interactions. Real-time monitoring of cell growth showed that MCF-7 cells treated with NDL exhibited a clear cytostatic profile, different from control cells, for more than 20 h. During this period, the four ingredients alone and three-ingredient combinations shared a kinetic profile similar to the control cells. This suggested a different mode of action of NDL other than what is exerted by the single ingredients and the three-ingredient combinations. As previously reported, biological activities of a herbal combination resulted from interactions among the different components rather than from activities of individual ingredients [18]. Moreover, the mode of action of a herbal formula could be more or less different from individual ingredients, expressed by different profiles of gene expression of treated cells [19].

In chemotherapy, high cytotoxicity toward cancer cells with minimal harm to normal cells is the ideal approach. The selectivity index (SI) reflects the differential cytotoxicity of a compound against tumor and normal cells. The greater the SI value of a compound, the more selective it is. An SI value above 2 indicates cytotoxic selectivity [20]. At the IC_50_ concentration, NDL cytotoxicity was significantly greater on MCF-7 and Hep G2 cells than on normal fibroblasts. NDL also exhibited higher cytotoxicity against NCI-H460 than fibroblasts, though the difference was not significant. The SI values of NDL, calculated from the cytotoxicity of NDL against normal fibroblasts and MCF-7, Hep-G2 and NCI-H460 were 6.45, 1.61, and 1.29, respectively, indicating a highly selective cytotoxic effect of NDL against MCF-7 cells and a general toxicity to Hep-G2 and NCI-H460 cells. The high cytotoxicity of NDL toward MCF-7 cells could be explained partly by the presence of flavonoids in the NDL ingredients (data not shown). The cytotoxicity of flavonoids on breast cancer cells, such as MCF-7 cells, is closely related to the expression of estrogen receptors [21]. In an ongoing study, we showed that NDL upregulated the expression of estrogen receptor beta (data not published), which was shown to inhibit human mammary epithelial cell growth [22]. Real-time monitoring of cell growth in the short term revealed a rapid cell index increase only on MCF-7 cells after treatment with NDL at a concentration of 4.3 mg/mL followed by rapid cell index decrease, corresponding to cell detachment. This kinetic profile resembled the profile generated by thapsigargin, a compound that modulates intracellular calcium level [23]. The increase of cytosolic calcium leads to transient changes in cell morphology, which explains the change of cell index value [24]. Polyphenolic compounds induce calcium release and disrupt mitochondrial function, leading to selective cytotoxicity toward MCF-7 cells [25]. Long-term monitoring of MCF-7 cell and fibroblast proliferation showed that they responded differently to the same concentrations of NDL. Fibroblasts maintained a better and longer survival rate compared to tumor cells. The antiproliferative effect of NDL was expressed through a cell cycle non-phase-specific as indicated by flow cytometry analysis. When cells were induced to a cytostatic status, they should resolve the situation either by death or by escape from the growth inhibitory pressure [26]. Treated fibroblasts can escape the cytostatic effect of NDL, but MCF-7 cells cannot and underwent apoptosis as a response to NDL treatment. Bioactive substances from each ingredient may contribute directly to the overall effect. Naringin, a flavonoid from mung bean, was reported to inhibit P-gp and breast cancer resistant protein, thus improving drug absorption [27]. Quercetin, found in mung bean seeds and sprouts, was shown to upregulate estrogen-binding sites type-II, thus exerting an inhibitory effect on MCF-7 cells even at low concentration [28]. Interestingly, naringenin, another flavonoid identified from mung bean, exerts cytotoxic effects on both estrogen receptor-positive and estrogen receptor-negative cells, which coexist in most breast cancers [21]. Breast cancer is the leading cause of cancer-related death among women around the world. Over the last two decades, breast cancer has become the most frequently diagnosed neoplasm in Vietnamese women [29]. A specific cytotoxicity toward MCF-7 cells of NDL could be an interesting perspective to be explored for breast cancer treatment.

Many Vietnamese plants are recognized as possessing anticarcinogenic, antiproliferative and cytotoxic activities. All ingredients of the NDL formula are used for both nutritional and medicinal purposes. A good perspective for investigating this formula was that its ingredients were easily obtainable at low cost, and could be ingested without severe toxicity to normal cells.

## Conclusions

VTM is a fully integrated part of Vietnamese health care system but its development is hampered by the lack of evidence-based support. Results from this study provided some scientific evidence for the cytotoxic effect of a formulation (NDL) composed of medicinal materials indigenous to Vietnam against a breast cancer cell line. The highly selective cytotoxicity of the NDL formula to MCF-7 cells may result from synergistic interactions of the four ingredients. We are currently working on determining the mechanism of action of the complete formula.

## Abbreviations

AO, acridine orange; CI: combinational index; EB, ethidium bromide; Fa: fraction affected; Inh %, percentage of growth inhibition; NDL, Nam Dia Long formula; Pa, *Pheretima aspergillum*; Sa, *Sauropus androgynous* (L.) Merr; SI, selective index; SRB, sulforhodamine B; Vr, *Vigna radiata* (L.) Wilczek; VTM, Vietnamese traditional medicine; Vu, *Vigna unguiculata* (L.) Walp. subsp. unguiculata
